# Follow‐up of late‐onset Pompe disease patients with muscle magnetic resonance imaging reveals increase in fat replacement in skeletal muscles

**DOI:** 10.1002/jcsm.12555

**Published:** 2020-03-04

**Authors:** Claudia Nuñez‐Peralta, Jorge Alonso‐Pérez, Jaume Llauger, Sonia Segovia, Paula Montesinos, Izaskun Belmonte, Irene Pedrosa, Elena Montiel, Alicia Alonso‐Jiménez, Javier Sánchez‐González, Antonio Martínez‐Noguera, Isabel Illa, Jordi Díaz‐Manera

**Affiliations:** ^1^ Radiology Department, Hospital de la Santa Creu i Sant Pau Universitat Autònoma de Barcelona Barcelona Spain; ^2^ Neuromuscular Disorders Unit, Neurology Department, Hospital de la Santa Creu i Sant Pau Universitat Autònoma de Barcelona Spain; ^3^ Centro de Investigación en Red en Enfermedades Raras (CIBERER) Barcelona Spain; ^4^ Philips Healthcare Iberia Madrid Spain; ^5^ Rehabilitation and Physiotherapy Department, Hospital de la Santa Creu i Sant Pau Universitat Autònoma de Barcelona Barcelona Spain; ^6^ John Walton Muscular Dystrophy Research Center University of Newcastle UK

**Keywords:** Enzymatic replacement therapy, Fatty replacement, Muscle degeneration, Muscle MRI, Muscle wasting, Pompe disease

## Abstract

**Background:**

Late‐onset Pompe disease (LOPD) is a genetic disorder characterized by progressive degeneration of the skeletal muscles produced by a deficiency of the enzyme acid alpha‐glucosidase. Enzymatic replacement therapy with recombinant human alpha‐glucosidase seems to reduce the progression of the disease; although at the moment, it is not completely clear to what extent. Quantitative muscle magnetic resonance imaging (qMRI) is a good biomarker for the follow‐up of fat replacement in neuromuscular disorders. The aim of this study was to describe the changes observed in fat replacement in skeletal muscles using qMRI in a cohort of LOPD patients followed prospectively.

**Methods:**

A total of 36 LOPD patients were seen once every year for 4 years. qMRI, several muscle function tests, spirometry, activities of daily living scales, and quality‐of‐life scales were performed on each visit. Muscle MRI consisted of two‐point Dixon studies of the trunk and thigh muscles. Computer analysis of the images provided the percentage of muscle degenerated and replaced by fat in every muscle (known as fat fraction). Longitudinal analysis of the measures was performed using linear mixed models applying the Greenhouse–Geisser test.

**Results:**

We detected a statistically significant and continuous increase in mean thigh fat fraction both in treated (+5.8% in 3 years) and in pre‐symptomatic patients (+2.6% in 3years) (Greenhouse–Geisser *p* < 0.05). As an average, fat fraction increased by 1.9% per year in treated patients, compared with 0.8% in pre‐symptomatic patients. Fat fraction significantly increased in every muscle of the thighs. We observed a significant correlation between changes observed in fat fraction in qMRI and changes observed in the results of the muscle function tests performed. Moreover, we identified that muscle performance and mean thigh fat fraction at baseline visit were independent parameters influencing fat fraction progression over 4 years (analysis of covariance, *p* < 0.05).

**Conclusions:**

Our study identifies that skeletal muscle fat fraction continues to increase in patients with LOPD despite the treatment with enzymatic replacement therapy. These results suggest that the process of muscle degeneration is not stopped by the treatment and could impact muscle function over the years. Hereby, we show that fat fraction along with muscle function tests can be considered a good outcome measures for clinical trials in LOPD patients.

## Introduction

Pompe disease is a rare autosomal recessive disorder produced by mutations in the *GAA* gene, which encodes the enzyme acid alpha‐glucosidase. This enzyme metabolizes glycogen to glucose inside lysosomes^1^. Consequently, mutations in the *GAA* gene lead to glycogen accumulation in numerous tissues, but clinical symptoms are primarily because of cardiac and skeletal muscle involvement.^2^

Pompe disease has two distinct phenotypes. The classic infantile phenotype is characterized by the onset of symptoms in the first months of life, with muscle weakness, hypertrophic cardiomyopathy, and respiratory insufficiency.^3^ Patients die early if they are not treated.^4^ In contrast, late‐onset Pompe disease (LOPD) is more heterogeneous in its clinical presentation.^5^, ^6^ Patients may present with asymptomatic hyperckemia or develop a slowly progressive weakness involving respiratory, axial, and limb muscles.^7^, ^8^

Enzymatic replacement therapy (ERT) with alglucosidase alfa (Myozyme®, Sanofi‐Genzyme, Cambridge, MA, USA) is accepted as the standard treatment for Pompe disease. Several open label studies have demonstrated its efficacy in maintaining muscle function in LOPD since the original clinical trial was published in 2010.^9^, ^10^, ^11^ However, a recent single‐centre follow‐up study suggests that the initial improvement lasts for up to 2 or 3 years and is followed by a progressive deterioration in skeletal and respiratory muscle function.^12^ Clinical trials with new therapeutic strategies, including improved enzymes or genetic therapies, are being designed and will probably start in the next few years.^13^, ^14^ These trials will probably rely on functional outcome measures to demonstrate their efficacy. The changes that can be found in common muscle function tests in LOPD patients treated with Myozime® for several years have not been clearly established. Functional tests have the disadvantage of being subject to motivation, are influenced by several external factors, and often display limited interoperator reproducibility.^15^ Moreover, the process of muscle degeneration and fatty substitution may have started without yet influencing the results of the muscle function tests. Therefore, there is a need for robust biomarkers sensitive to disease progression and treatment efficacy.^16^

In recent years, muscle magnetic resonance imaging (MRI) has been proposed as a valuable tool for both the diagnosis and follow‐up of patients with muscle disorders. The identification of muscles replaced by fat can be easily assessed using T1‐weighted sequences, which is helpful in the diagnosis process of patients.^17^ However, patient follow‐up requires a more specific technique, such as the Dixon MRI sequence, which is able to quantify the amount of fat present in an area of skeletal muscle of interest.^18^ This test is useful in natural history studies or clinical trials. In the case of Pompe disease, two studies applied Dixon to a large cohort of LOPD patients and in both cases demonstrated that the sequence is able to identify an increase in fat replacement in skeletal muscles over a 1 year period.^19^, ^20^ However, it is not known if the rate of progression of fat replacement in LOPD is stable over time or which clinical and/or demographic factors can influence this progression.

The aim of our study was to describe the changes observed in fat replacement in skeletal muscles over a period of 4 years in a long cohort of LOPD patients.

## Methods

### Study design and participants

This study was a prospective, open‐label cohort study involving 36 patients with a confirmed diagnosis of LOPD performed at the Hospital de la Santa Creu i Sant Pau (HSCSP) in Barcelona, from December 2013 to June 2018. All patients were evaluated once every 12 months (±2 months) for a total of four visits. At each visit, we performed muscle function tests, spirometry, quality‐of‐life scales, and quantitative muscle MRI. The study was registered in ClinicalTrials.gov with the identifier NCT01914536. The HSCSP ethics committee approved the study, and all participants signed an informed consent form. All study procedures were performed in accordance with Spanish regulations.

Inclusion criteria for the study were (i) diagnosis of LOPD based on recommendations proposed by the European Pompe Consortium, reduced enzymatic activity in leukocytes, fibroblasts, or skeletal muscle and/or the presence of two mutations in the *GAA* gene^21^; (ii) no contraindications to MRI; and (iii) willingness to complete all muscle function tests, respiratory assessment, and patient‐reported outcomes measures. We included in the study both patients with skeletal muscle weakness and respiratory involvement and patients without symptoms.

All patients were studied by three physiotherapists (I.B., I.P., and E.M) with considerable experience in neuromuscular disorders at HSCSP. All patients were evaluated by the same physiotherapist throughout the whole study. The physiotherapists evaluated muscle function using the following tests: the 6 minute walking test, time to walk 10 m, timed up‐and‐go test, time to climb up and down four steps, and the Motor Function Measure 20‐item scale (MFM‐20). All timed tests were performed asking the patient to not use aids for walking. Muscle strength was studied using both the Medical Research Council (MRC) scale and hand‐held myometry. Daily life activities were studied using the activity limitations scale for patients with upper and/or lower limb impairments, and quality of life was analysed using both the Individualized Neuromuscular Quality of Life Questionnaire and the Short Form 36 questionnaire. We obtained forced vital capacity, both seated and lying down, using the Carefusion Microlab ML 3500 MK8 spirometer (Carefusion, Yorba Linda, CA, USA).

### Muscle imaging

All patients were examined in a 1.5 magnetic resonance system (1.5 Achieva dStream; Philips, Eindhoven, NL) at HSCSP as previously described.^22^ We used the same positioning protocol for all patients: a supine position with the legs stretched out.

Axial 3D Dixon Fast Field Echo was performed on the pelvis and thighs by using a thirty‐two body coil, with the following parameters: the repetition time (TR) the echo time (TE) = 5.78/1.8, 4 m, flip angle = 15°, field of view = 520 × 340 × 300 mm, voxel size = 1 × 1 × 3 mm for thighs, and field of view = 520 × 320 × 200 mm and voxel size = 1.3 × 1.7 × 5 mm for pelvis. This Dixon sequence follows a seven peak fat modelling and provides separate images for fat and water. The acquisition time was 45 min per patient.

Three investigators (C.N‐P., A.A‐P, and J.D‐M.) analysed the Dixon MR images using a Philips Research Image Development Environment tool developed for this project. Regions of interests (ROIs) were manually drawn on four slices of the following muscles: *rectus femoris*, *vastus intermedius*, *vastus lateralis*, *vastus medialis*, *adductor magnus*, *sartorius*, *gracilis*, *semitendinosus*, and *semimembranosus*; on three slices of *biceps femoris long head, biceps femoris short head*, and *adductor longus*; and on one slice for psoas and lumbar paraspinal muscles. Slices covered the proximal, middle, and distal part of the muscles. We used anatomical landmarks to ensure consistency between annual analyses. For every ROI, the total area and area covered by fat were calculated automatically using the Philips Research Image Development Environment tool. The fat fraction (FF) coefficient was defined as fat/(fat + water), where fat and water were the image intensity values over the ROI for the fat and water Dixon images, respectively. From those two parameters, and assuming that water content corresponds mainly to muscle, fat and muscle areas were estimated. Cumulative values across all slices were also computed, and as a final index, muscle FF was calculated as follows: FF = (muscle fat area × 100)/muscle area. Once we obtained the values from every muscle, we calculated the thigh FF as follows: thigh FF = (sum of fat area of all thigh muscles × 100)/sum of muscle area of all thigh muscles. For every muscle, we calculated the muscle fatty transformation rate = FF final visit − FF initial visit/(one‐initial FF).

The time required to quantify FFs in Dixon images was 45 to 60 min per patient. A high degree of reliability was found between investigators. The Intraclass coefficient (ICC) coefficient was 0.982, with a 95% confidence interval from 0.977 to 0.987.

### Statistics

We confirmed that the variables were normally distributed using a Kolmogorov–Smirnov test, and therefore, parametric statistical studies were used. Longitudinal analysis of the different outcome measures, including muscle function tests and muscle FF quantified by MRI, was performed using linear mixed models applying the Greenhouse–Geisser test. To evaluate the magnitude of the changes observed in the results of outcome measures over the 3 year follow‐up period, we provide the standardized response mean (SRM). The SRM is calculated by dividing the mean score change (final visit minus initial visit) by the standard deviation of the change. The estimation of the magnitude of change over time using this formula is based on Cohen's rule for effect size (ES): ‘trivial’ (ES < 0.20), ‘small’ (ES ≥ 0.20 <. 50), ‘moderate’ (ES ≥ 0.50 < 0.80), or large (ES ≥ 0.80).^22^

To study the influence of clinical and demographic factors in the progression of skeletal muscle FFs, we performed a two‐step process. In the first exploratory step, we used a Pearson test to analyse the correlation between every factor of interest and the increase in thigh FF. Those factors with both a statistically significant correlation and a correlation coefficient higher than 0.4 were selected for the second step. This second step consisted of an ANCOVA analysis in which the influence of every selected factor on mean thigh FF increase, as detected by MRI, was assessed separately. The significance level for all statistical studies was set at *p* < 0.05, and post hoc Bonferroni corrections were used when needed. Statistical studies were performed with SPSS for Mac computers (Version 21, SPSS Inc, Chicago, IL). Heatmaps were developed using r version 3.1.1 (R Foundation for Statistical Computing, Vienna, Austria).

## Results

### Description of the cohort

Thirty‐six LOPD patients (20 women, 55.5%) were enrolled in this study. Mean age at the start was 43.9 ± 14.8 years old. Table 1 provides the main clinical data of the patients at visit 0 (baseline). At Visit 0, 23 patients were already being treated with ERT with Myozyme® for a mean period of 4.1 years. All these patients continued the treatment without interruptions during the whole follow‐up. Out of the 13 remaining patients, four started treatment during the follow‐up because of the development of muscle weakness impairing the flexion and/or extension of the hip, as demonstrated in clinical examination. These four patients have been excluded from the statistical studies, and their results are shown separately. All these patients started treatment 1 to 3 months after Visit 1. The remaining nine patients were considered pre‐symptomatic because they did not report any relevant symptoms or had no muscle weakness found by clinical examination, at any time during the study. At Visit 0, 10 patients needed walking aids such as a cane or a stick for walking outdoors, and 11 patients needed nocturnal non‐invasive ventilation support (*Table* 1). During the follow‐up period, three patients started using walking aids, and two more started using non‐invasive ventilation at night.^20^

**Table 1:** Clinical features of the patients included in the study

P	G	Age at onset symptoms (years)	Age at ERT (years)	Age at Visit 0 (years)	Baseline walking aids	Baseline ventilation	Last visit walking aids	Last visit ventilation
1	F	38	47	50	N	N	Y (stick)	N
2	F	30	39	48	Y (stick, wheelchair)	Yes (non‐invasive)	Y (fully wheelchair)	Yes (non‐invasive)
3	F	—	—	26	N	N	N	N
4	F	49	59	63	Y (crouch)	N	Y (crouch)	N
5	F	30	42	45	N	N	N	N
6	F	27	47	51	N	N	N	N
7	M	62	67	66	N	Y (non‐invasive)	N	Y (non‐invasive)
8	F	48	52	59	Y (stick)	N	Y (stick)	Y (non‐invasive)
9	F	40	48	55	N	N	N	N
10	M	36	39	42	N	Y (non‐invasive)	N	Y (non‐invasive)
11	F	15	24	31	Y (Walker, wheelchair)	Y (non‐invasive)	Y (Walker, wheelchair)	Y (non‐invasive)
12	F	27	39	46	N	N	N	N
13	M	35	45	47	N	Y (non‐invasive)	N	Y (non‐invasive)
14	M	41	45	51	N	Y (non‐invasive)	N	Y (non‐invasive)
15	F	39	46	51	Y (crouch)	Y (non‐invasive)	Y (crouch)	Y (non‐invasive)
16	M	—	—	22	N	N	N	N
17	M	—	—	51	N	N	N	N
18	M	—	—	14	N	N	N	N
19	F	40	64	65	Y (stick)	N	Y (stick)	N
20	F	24	29	35	Y (crouch)	N	Y (crouch)	N
21	F	36	40	41	N	Y (non‐invasive)	N	Y (non‐invasive)
22	F	14	45	52	N	N	Y (stick)	N
23	M	22	57	64	Y (Stick)	Y (non‐invasive)	Y (stick)	Y (non‐invasive)
24	M	—	—	8	N	N	N	N
25	F	42	55	57	Y (Stick)	Y (non‐invasive)	Y (stick)	Y (non‐invasive)
26	M	35	43	46	N	Y (non‐invasive)	N	Y (non‐invasive)
								
27	M	34	51	51	N	N	N	Y (non‐invasive)
28	M	—	—	51	N	N	N	N
29	M	32	43	44	N	N	N	N
30	F	39	48	54	N	N	Y (stick)	N
31	M	—	—	12	N	N	N	N
32	M	—	—	51	N	N	N	N
33	M	38	43	42	Y (stick)	N	Y (stick)	N
34	F	20	22	21	N	N	N	N
35	F	—	—	35	N	N	N	N
36	F	24	40	29	N	N	N	N

F, female; G, gender; M, male; N, not needed; P, patient number; Y, yes.

### Functional assessments

The muscle function tests that showed significant differences in symptomatic‐treated patients after the follow‐up period were lower limb muscle strength, measured using MRC and hand‐held myometry, and respiratory muscle strength (forced vital capacity seated), with *p* values lower than 0.01 and high SRMs as shown in *Table* 2. Specific details of the myometry assessments are detailed in *Table* 3. In summary, all myometry assessments of the lower limbs but knee flexion showed worsening of muscle strength, with knee extension and hip flexion being the two that worsened the most (−4.9 and −4.3 kg, respectively). In contrast, myometry assessments of the upper limbs did not show significant differences. We did not observe significant differences in timed tests, MFM‐20, or daily life activity scales after this period of time. In the case of pre‐symptomatic patients, we did not observe significant differences in any of the results of the motor function tests, spirometry, or daily life activity scales.

**Table 2:** Change between Visits 0 and 3 evaluation in muscle function tests, spirometry, quantitative muscle magnetic resonance imaging, and patient reported outcome measures in treated and pre‐symptomatic late‐onset Pompe disease patients

Test	Symptomatic treated patients *n* = 22				Pre‐symptomatic patients *n* = 9			
	Visit 0	Visit 3	P	SRM	Visit 0	Visit 3	P	SRM
Time to walk 10 m (s)	7.9 ± 3.8	7.8 ± 6.1	0.30	0.3	3.1 ± 0.5	2.8 ± 0.8	0.39	0.1
6MWT (m)	394.4 ± 150.4	422.3 ± 140.1	0.16	0.1	599.6 ± 85.4	596.2 ± 69.5	0.29	0.03
Timed up & go (s)	7.5 ± 7.2	10.2 ± 6.2	0.20	0.4	3.8 ± 1.5	4.1 ± 0.9	0.44	0.4
Time to climb up four stairs (s)	4.8 ± 3.5	4.7 ± 2.9	0.59	0.3	1.4 ± 0.3	1.3 ± 0.3	0.20	0.3
Time to go down four stairs (s)	3.6 ± 2.4	3.3 ± 2.1	0.40	0.3	1.3 ± 0.1	1.3 ± 0.2	0.61	0.3
MRC total score	95.5 ± 11.1	90.8 ± 11.8	0.0001	0.9	110 ± 0	110 ± 0	0.99	0
MRC LL score	47.1 ± 10.2	42.5 ± 10.3	0.001	0.8	60 ± 0	60 ± 0	0.99	0
Myometry total (kg)	203.7 ± 118.8	180.9 ± 107.7	0.005	0.6	312.3 ± 91.3	354.9 ± 82.3	0.21	0.2
Myometry LL (kg)	117.5 ± 67.7	97.3 ± 58.3	0.0001	0.8	172.3 ± 81.6	201.6 ± 47.0	0.35	0.3
Myometry UL (kg)	95.2 ± 50.4	91 ± 48.4	0.27	0.2	130 ± 10.2	145.8 ± 9.4	0.08	0.5
MFM20 score	48 ± 8	47.6 ± 8.7	0.32	0.1	59 ± 2.4	59 ± 1.9	0.66	0
FVC seated (L)	75.9 ± 22.9	69 ± 25.4	0.0001	0.8	93.6 ± 12.8	90.1 ± 10.3	0.48	0.2
FVC supine (L)	67.6 ± 25.3	65.7 ± 24.1	0.74	0.4	89.3 ± 17.6	88.3 ± 22.5	0.37	0.1
ACTIVLIM score	28 ± 7	27.7 ± 6.8	0.84	0.1	36 ± 0	36 ± 0	0.99	0
Mean thigh FF (%)	36.5 ± 18.6	42.31 ± 19.39	0.0001	1.6	11.9 ± 4.4	15.1 ± 5.1	0.01	1.2
Mean Ps FF (%)	81.8 ± 10.2	82.9 ± 8.6	0.32	0.3	38.3 ± 24.4	45.8 ± 24.3	0.26	0.6

Mean value and standard deviation are shown. Greenhouse–Geisser test analysing the data from Visits 1, 2 and 3 were used to find out whether the differences observed were statistically significant.

ACTIVLIM, activity limitations; FF, fat fraction; FVC, forced vital capacity; LL, lower limbs; MFM20, Motor Function Measure 20‐item scale; MRC, Medical Research Council; Ps, paraspinal muscles; SRM, standardized response mean; UL, upper limbs; 6MWT, 6 min walking test.

**Table 3:** Change between Visits 0 and 3 evaluation in hand‐held myometry assessments in symptomatic‐treated and pre‐symptomatic late‐onset Pompe disease patients

Test	Symptomatic treated patients *n* = 22				Pre‐symptomatic patients *n* = 9			
	Visit 0	Visit 3	*p*	SRM	Visit 0	Visit 3	*p*	SRM
Neck flexion	7.3 ± 5.3	8.1 ± 6.1	0.1	0.15	15.5 ± 5.7	15.1 ± 6.7	0.2	−0.05
Shoulder abduction	23.2 ± 10.3	22.4 ± 10.2	0.52	−0.12	27.6 ± 9.7	27.7 ± 6.7	0.98	0.01
Shoulder adduction	15.4 ± 10.3	16.2 ± 2.2	0.37	0.14	26.5 ± 8.7	29.9 ± 7.8	0.02	0.37
Elbow flexion	25.3 ± 12.1	22.9 ± 2.3	0.06	−0.38	29.7 ± 9.4	37.4 ± 7.7	0.02	0.82
Elbow extension	19.2 ± 9.9	17.8 ± 8.2	0.13	−0.34	24.2 ± 7.4	26.3 ± 6.2	0.22	0.41
Hip flexion	21.4 ± 13.8	17.1 ± 12.7	**0.007**	−0.61	33.9 ± 11.9	32.5 ± 12.3	0.46	−0.5
Hip extension	11.3 ± 9.1	8.7 ± 8.9	**0.001**	−0.76	33.2 ± 25.1	35.1 ± 8.1	0.06	0.19
Hip abduction	18.3 ± 12.1	14.6 ± 10.9	**0.005**	−0.63	33.4 ± 17.7	30.3 ± 10.7	0.46	−0.24
Hip adduction	11.5 ± 7.3	8.8 ± 6.3	**0.001**	−0.76	20.8 ± 6.3	19.8 ± 5.7	0.27	−0.42
Knee extension	33.6 ± 19.8	28.7 ± 16.4	**0.01**	−0.54	48.1 ± 15.7	47.1 ± 11.5	0.88	−0.06
Knee flexion	20.1 ± 14.2	19.1 ± 11.4	0.31	−0.18	35.2 ± 13.2	34.7 ± 8.2	0.91	−0.04

Mean value and standard deviation are shown. Greenhouse–Geisser test analysing the data from Visits 1, 2, 3 and 4 was used to find out whether the differences observed were statistically significant, and *p* values are shown. All units are expressed as kilogrammes.

SRM, standardized response mean.

Bold emphasis mean statistical significance

### Quantitative muscle magnetic resonance imaging: progression of fat fraction in late‐onset Pompe disease patients

**Figure 1:**
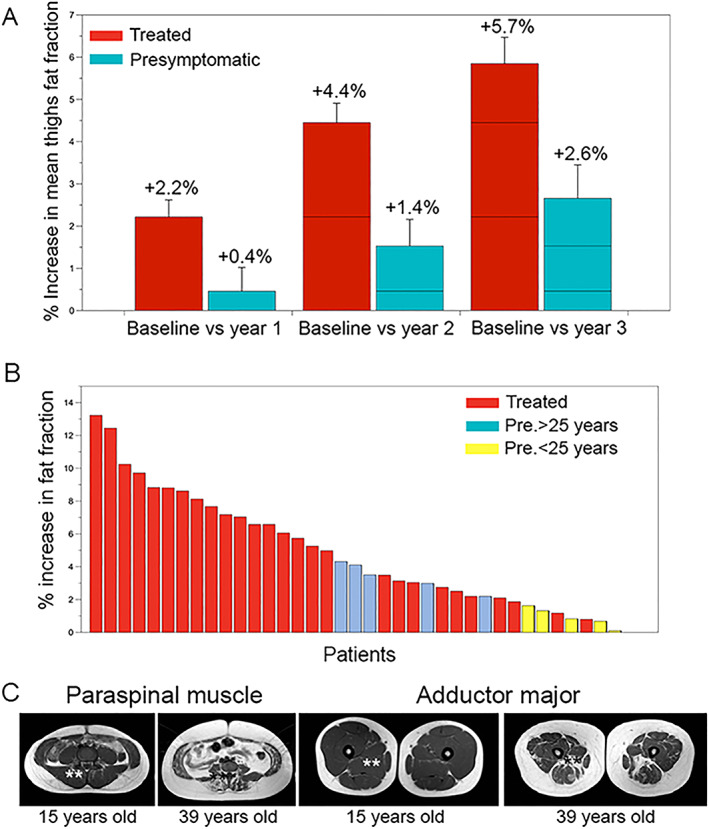
(A) Increase in mean thigh fat fraction at year 1, year 2 and year 3 related to the baseline value. Red bars show data from symptomatic patients treated while blue bars show data from presymptomatic patients. Mean increase and standard error is provided. (B) Increase in mean thigh fat fraction in every single patient of the cohort at visit 3 related to baseline. Red bars represent ERT treated patients. Blue bars represent presymptomatic non‐treated patients older than 25, and yellow bars represent patients younger than 25 years old. (C) Involvement of paraspinal and adductor major muscles in presymptomatic patients depending on the age. Fat replacement and atrophy of both muscles was observed in patients older than 25 years old.  *Paraspinal muscles **Adductor major muscles

**Figure 2:**
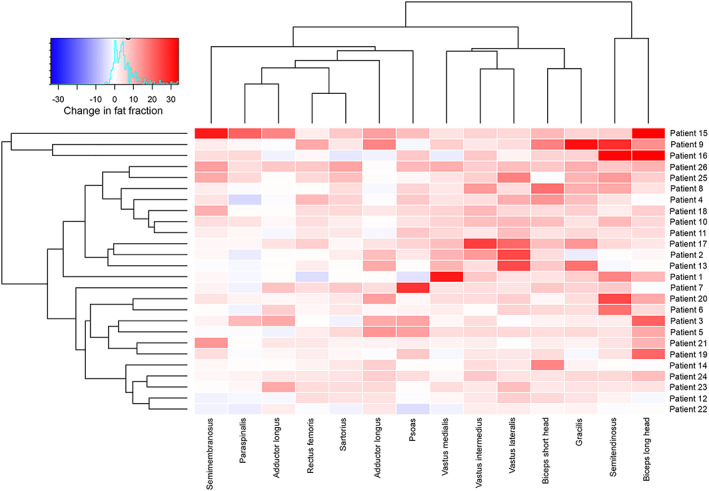
Heatmaps showing changes in fat fraction of thigh muscles studied in symptomatic‐treated late‐onset Pompe disease patients. Patients and muscles are ordered according to hierarchical clustering with increasing replacement severity from bottom to top (patient‐rows) and from left to right (muscles‐columns). The increase in fat fraction over a 3 year period of time in the muscle of a patient is indicated by the colour of the square. Red colours mean increased fat fraction, while blue colours mean decreased fat fraction.

**Figure 3:**
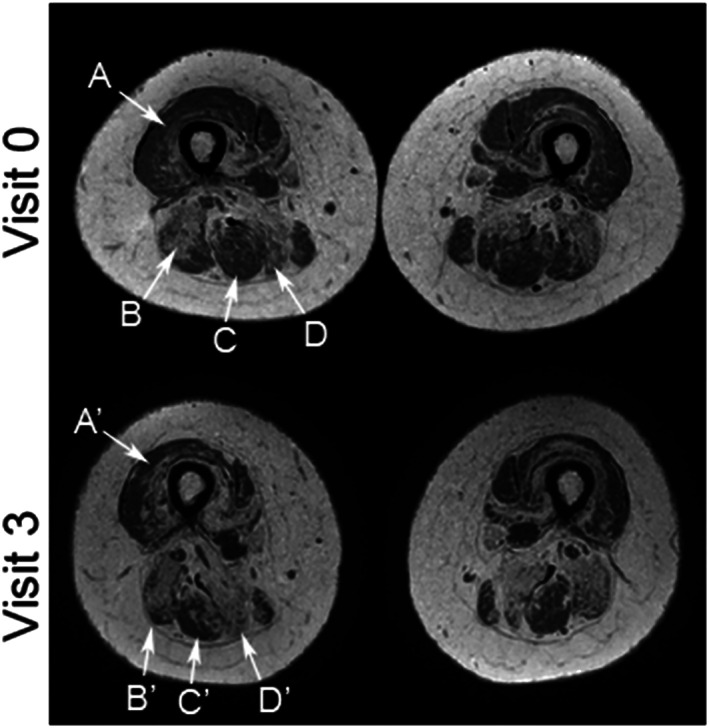
Images illustrate the change in fat replacement throughout the follow‐up. Fat fraction maps acquired from the thigh at Visits 0 and 3 (0–100% scale). Increase in fat replacement was visible in most of the muscles, with changes most obvious in the following muscles: (A and A') vastus lateralis (in this patient fat fraction increased from 15.5% to 35.7%), (B and B') long head of the biceps femoris (fat fraction increased from 57.6% to 79.4%), (C and C') semitendinosus (fat fraction rose from 20.3% to 51.4%), and (D and D') semimembranosus (fat fraction increased from 74.2% to 82.4%).

We performed a total of four visits for the whole cohort of patients using two‐point Dixon. In every case, we calculated the area of the muscle, the muscle fraction, and the FF of psoas, paraspinal muscles, and each muscle in the thighs, and from that, we derived the mean thigh FF. In the case of symptomatic patients treated with Myozyme®, we observed a statistically significant increase in mean thigh FF of 5.7 ± 3.7% at the end of the follow‐up, which corresponds to an increase of 1.9% per year (Greenhouse–Geiser test, *p* < 0.0001 and SRM 1.6) (*Figure* 1A). Increase in mean thigh FF varied substantially from one patient to another, with a maximum of +13.2% and a minimum of +0.8% (*Figure* 1B). We observed a significant increase in FF in all muscles analysed, except the paraspinal muscle, as described in *Table* 4 and displayed in *Figure* 2. An example of this increase can be found in *Figure* 3.

**Table 4:** Increase in fat fraction described by each muscle analysed in treated and pre‐symptomatic patients

	Symptomatic treated patients (*n* = 23)				Pre‐symptomatic patients (*n* = 9)			
Muscle	Visit 0 FF	Visit 3 FF	P	SRM	Visit 0 FF	Visit 3 FF	P	SRM
Rectus femoris	19.5 ± 18.7	21.1 ± 17.1	**0.001**	0.84	8.8 ± 2.6	11.3 ± 2.7	**0.007**	1.02
Vastus medialis	31.64 ± 26.9	31.35 ± 27.1	**0.009**	0.71	10.2 ± 2.2	12.3 ± 2.4	0.14	1.01
Vastus lateralis	22.6 ± 16.4	27.2 ± 21.1	**0.0001**	1.07	8.5 ± 1.8	12 ± 2.6	**0.007**	1.34
Vastus intermedius	38.7 ± 27.2	40.6 ± 28.5	**0.0001**	1.14	9.7 ± 3.1	14.3 ± 4.1	**0.01**	1.76
Biceps short head	28.3 ± 20.5	32.7 ± 21.5	**0.07**	1.18	12.8 ± 4.3	15.7 ± 4.2	0.33	0.61
Biceps long head	55.8 ± 29.1	64 ± 26	**0.0001**	0.96	11.8 ± 5.1	15.4 ± 7.2	**0.03**	2.91
Semimembranosus	66.7 ± 26.4	70 ± 24	**0.02**	0.63	16.9 ± 12.7	21.6 ± 14.5	0.69	0.47
Semitendinosus	46.7 ± 29.1	51.3 ± 32.8	**0.0001**	0.98	12 ± 4.2	14.2 ± 5.8	0.14	1.12
Adductor major	79.4 ± 17.2	79.2 ± 17.3	**0.02**	0.69	22.1 ± 20.1	27.2 ± 26.3	0.37	0.69
Adductor longus	51.7 ± 33.6	51.9 ± 32.5	**0.0001**	0.86	11 ± 3.2	14.8 ± 3.2	**0.01**	3.43
Sartorius	25.3 ± 17.6	27.8 ± 17	**0.0001**	0.89	19.8 ± 5.3	20.7 ± 5.5	0.49	0.02
Gracillis	22.8 ± 13	27 ± 12.9	**0.0001**	0.91	15.2 ± 3.8	17. 2 ± 4.4	0.27	0.4
Paraspinalis	80.4 ± 9.7	83.3 ± 6	0.29	0.3	38.3 ± 24.4	45.8 ± 24.3	0.26	0.6
Psoas	54.8 ± 30.6	60.6 ± 28.7	0.051	0.22	24.7 ± 16.2	29.2 ± 15.9	0.27	0.26
Total thighs	39.5 ± 18.2	42.6 ± 19	**0.0001**	1.69	12.1 ± 4.2	15.1 ± 5	**0.0001**	1.26

Mean fat fraction and standard deviation at Visits 0 (baseline) and 3 (Year 3) are provided. Linear mixed model applying Greenhouse–Geisser test was performed, and *p* is provided. Bold numbers in *p* values are related with significant changes. The standardized response mean (SRM) for every muscle is also provided.

FF, fat fraction.

In the case of pre‐symptomatic patients, we observed a statistically significant increase in mean thigh FF of 2.6 ± 1.6% at the end of the follow‐up, which represents an increase of 0.8% per year (Greenhouse–Geiser test, *p* < 0.0001) (*Figure* 1B). However, we identified higher values of FF in the *adductor major* and the paraspinal muscles of some pre‐symptomatic patients at Visit 0, especially those older than 25 (*Figure* 1D). In fact, we observed a positive correlation between the age at study of pre‐symptomatic patients, and the percentage of FF in these two muscles (adductor major: *ρ* = 0.71, *p* = 0.02; paraspinal muscles: *ρ* = 0.96, *p* = 0.0001; Pearson test). On the basis of these data, we decided to divide our cohort of pre‐symptomatic patients into younger than 25 years old at baseline (*n* = 5 patients) and older than 25 years old at baseline (*n* = 4 patients). Mean thigh FF did not change significantly in pre‐symptomatic patients younger than 25 (+0.7 ± 0.3% in 4 years; Greenhouse–Geisser *p* = 0.33), while it increased significantly in patients older than 25 (+3.2 ± 0.4% in 4 years; Greenhouse–Geisser *p* = 0.004).

### Correlation between changes in muscle function and fat fraction identified by magnetic resonance imaging

We observed a significant correlation between increase in mean thigh FF and changes in muscle strength as measured by hand‐held myometry and MRC, as shown in *Table* 5. The strongest correlation was between increase in FF in the *vasti* muscles and decrease in muscle strength for knee extension (*ρ* = 0.54, *p* = 0.02). We also found significant correlations between increase in mean thigh FF and the changes observed in the following timed tests: time to walk 10 m, 6 min walking test, time to climb up four steps and time to go down four steps (*Table* 5). In contrast, we did not identify a significant correlation between increase in mean thigh FF and results of the MFM‐20 score or the activity limitations test.

**Table 5:** Correlation between increase in fat fraction during the follow‐up and changes observed in muscle function tests in late‐onset Pompe disease patients (*n* = 32)

Variable	Significance (*p* value)	Correlation coefficient
**Time to walk 10 m (s)**	**0.011**	**0.43**
**6MWT (m)**	**0.024**	**‐0.48**
Timed up & go (s)	0.09	0.32
**Time to climb four stairs (s)**	**0.017**	**0.46**
**Time to go down four stairs (s)**	**0.015**	**0.47**
MRC LL score (kg)	0.31	0.15
**Myometry LL (kg)**	**0.032**	**‐0.4**
MFM‐20 (score)	0.56	‐0.13
**FVC seated (L)**	**0.023**	**‐0.39**
FVC supine (L)	0.20	‐0.24
ACTIVLIM score	0.29	0.18

Correlation was studied using Pearson test. *p* value was considered significant if lower than 0.05.

ACTIVLIM, activity limitations; FVC, forced vital capacity; LL, Lower limbs; MFM20, Motor Function Measure 20‐item scale; MRC, Medical Research Council; 6 min walking test.

Bold emphasis means variables that were selected for the ANCOVA study

### Analysis of patients who started treatment during follow‐up

A total of four patients started treatment with Myozyme® during follow‐up, because of the onset of muscle weakness. All cases started the treatment shortly after Visit 1 (1 to 3 months later). *Table* 6 shows the mean thigh FF at Visit 0 (baseline) and the changes observed at every visit in these four patients. We identified a reduction in the rate of FF increase between Visits 0 and 1 (before treatment was started, mean increase +3.39%), Visits 1 and 2, and Visits 2 and 3 (both after starting the treatment, mean increase +1.52% and +1.71% respectively). However, we did not apply any statistical tests to these data because they only correspond to four patients and can therefore be considered merely descriptive, requiring further evaluation.

**Table 6:** Changes in mean thighs fat fraction observed in the four patients who started the treatment during the follow‐up due to the presence of muscle weakness

Patient	Mean thighs fat fraction			
	Visit 0 (%)	Visit 1 (%)	Visit 2 (%)	Visit 3 (%)
Patient 1	25.26	30.66, +5.4	31.74, +1.08	32.25, +0.51
Patient 2	9.85	9.43, −0.42	9.02, ‐0,41	10.53, +1.51
Patient 3	57.69	62.11, +4.42	65.5, +3.39	67.93, +2.43
Patient 4	26.05	30.22, +4.17	32.27, +2,05	34.67, +2.4
Group		+3.39	+1.52	+1.71

Mean thighs fat fraction and increase over previous visit is shown for every patient.

### Factors influencing increase in fat fraction

**Figure 4:**
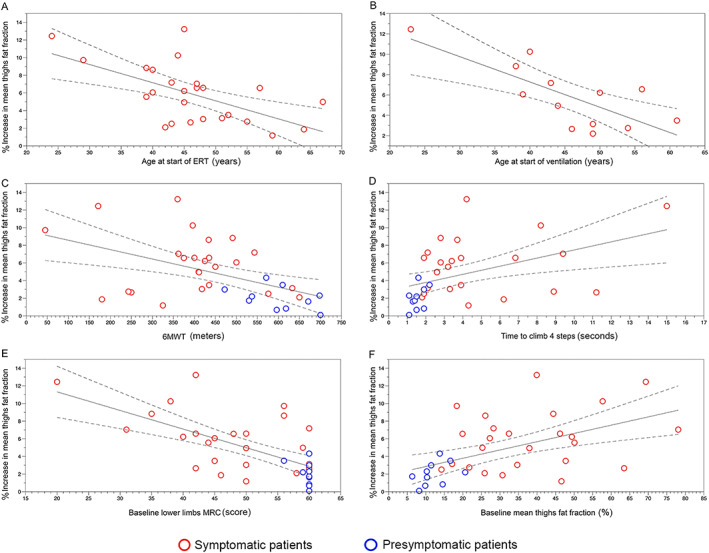
(A) Correlation between age at start of ERT and increase in mean thigh fat fraction. (B) Correlation between age at start of ventilation and increase in mean thigh fat fraction. (C) Correlation between results of the 6 min walking test (6MWT) at baseline and increase in mean thigh fat fraction. (D) Correlation between time to climb four steps at baseline and increase in mean thigh fat fraction. (E) Correlation between MRC for lower limbs at baseline and increase in mean thigh fat fraction. (F) Correlation between mean thigh fat fraction at baseline and increase in mean thigh fat fraction. ERT, enzymatic replacement therapy; MRC, magnetic resonance imaging.

We analysed which demographic, clinical, and muscle‐functional factors could influence the progression of mean thigh FF in Pompe disease patients. In a first step, we studied which variables had a significant correlation with mean increase in thigh FF, using Pearson correlation tests (*Table* 7). After that, in a second step, we confirmed which of these variables were influencing progression of FF using an ANCOVA analysis. *Table* 8 shows the variables that correlated with the progression of FF in our cohort of patients after this analysis, and *Figure* 4 displays the graphics showing correlations between variables. We observed that at an earlier start of the symptoms, a longer period of treatment, muscle function at baseline, and muscle FF at baseline were associated with a higher increase in FF in the muscle MRI.

**Table 7:** Correlation between increase in fat fraction after 4 years of follow‐up and demographic, clinic, and muscle function tests at baseline visit

Variable	Significance (*p* value)	Correlation coefficient
Sex (M/W)	0.43	−0.15
Age at onset of symptoms (year)	0.14	0.12
Time of progression (year)	**0.049**	0.34
Age at onset of ERT (year)	**0.002**	−0.7
Time on ERT (year)	**0.019**	0.49
Antibody titers	0.87	0.05
Use of aids for walking (Yes/No)	0.31	0.15
Use of ventilation (Yes/No)	**0.016**	0.42
Time to walk 10 m (s)	0.06	0.37
6MWT (m)	**0.005**	0.52
Time to climb four steps (s)	**0.004**	0.53
Time to go down four steps (s)	**0.07**	0.35
Timed up & go test (s)	0.38	0.22
MFM20 (score)	**0.002**	−0.57
MRC lower limbs (score)	**0.0001**	−0.65
Myometry lower limbs (kg)	0.1	−0.39
FVC (L)	0.73	0.15
ACTIVLIM (score)	0.65	0.16
Baseline mean thigh FF (%)	**0.001**	0.58

Correlation was studied using Pearson test. Antibody titers were studied at baseline visit.

ACTIVLIM, activity limitations; ERT, enzymatic replacement therapy; FVC, forced vital capacity; M, men; MFM20, Motor Function Measure 20‐item scale; MRC, Medical Research Council; 6 min walking test; W, women.

Bold emphasis:patients with statistical significance

**Table 8:** A one‐way analysis of covariance was conducted to determine which factors influenced the progression of mean thighs fat fraction

Factor	Effect on increase in FF
Time of progression	0.49
**Age at onset of ERT**	**0.001**
**Time on ERT**	**0.045**
Ventilation	0.56
**Age at ventilation**	**0.007**
Time to walk 10 m	0.052
**6MWT**	**0.009**
**Time to climb four steps**	**0.004**
Time to go down four steps	0.12
**MRC lower limbs**	**0.0001**
Myometry lower limbs	0.21
**MFM20**	**0.001**
**Baseline FF**	**0.0001**

Results of Greenhouse–Geisser test are shown and were considered significant if *p* < 0.05.

ERT, enzymatic replacement therapy; FF, fat fraction; MFM20, Motor Function Measure 20‐item scale; MRC, Medical Research Council; 6 min walking test.

Bold emphasis: statistical significance

### Muscle fat fraction progression: recommendations for the follow‐up

**Figure 5:**
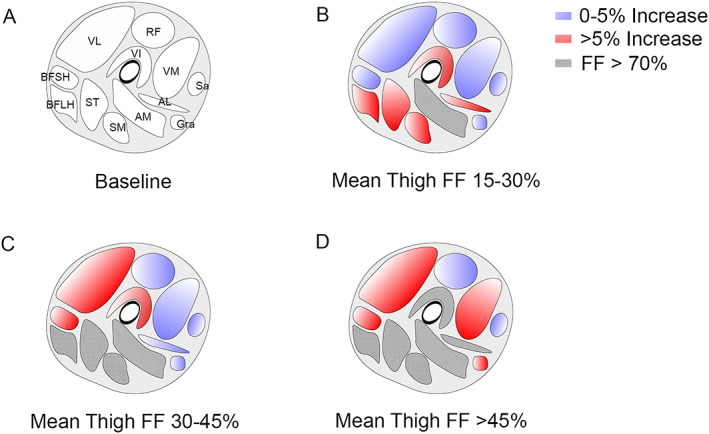
Progression of fat fraction depending on the baseline mean thigh fat fraction. (A) Baseline image showing the localization of thigh muscles (VL: *vastus lateralis*, RF: *rectus Femoris*, VM: *vastus medialis*, VI: *vastus intermedius*, BFSH: *biceps femoris short head*, BFLH: *biceps femoris long head*, ST: *semitendinosus*, SM: *semimembranosus*, AM: *adductor major*, AL: *adductor longus*, Sa: *sartorius*, and Gra: *gracilis*). (B) Increase in fat fraction over a 4 year period in patients with baseline mean thigh fat fraction of 15% to 30%. (C) Increase in fat fraction over a 4 year period in patients with baseline mean thigh fat fraction of 30% to 45%. (D) Increase in fat fraction over a 4 year period in patients with baseline mean thigh fat fraction >45%. FF, fat fraction.

We wanted to identify which muscles could be best for following up fatty replacement in LOPD patients in clinical trials or natural history studies. Increase in FF in most of the muscles analysed was statistically significant in symptomatic‐treated patients at the end of the follow‐up period. However, we observed a high variability in the amount of increase from one patient to another. In order to classify patients in similar groups, we divided our cohort into four groups, depending on the mean thigh FF at Visit 0 (baseline), which we had previously described as correlating with the clinical situation of the patient.^23^ *Table* 9 shows the four groups and the changes observed in FF during the follow‐up. The first group was ‘baseline FF lower than 15%’. All individuals in that group (*n* = 9) were pre‐symptomatic. Although these patients had never had muscle weakness or any symptoms of muscle weakness, we already detected high values of FF in the paraspinal muscles at baseline visit (FF = 35%). In this group, the muscle with a higher progression was the paraspinal muscles. The second group (15% to 30% baseline mean thigh FF) included 11 symptomatic patients. In this group, both paraspinal and *adductor major* were the two muscles showing the highest values of FF at baseline (78.8% and 75.3% respectively). The muscle with a more uniform progression was the long head of the *biceps femoris long head*. The third group (31% to 45% baseline mean thigh FF) included six individuals, all of whom were symptomatic. In this group, paraspinal, *adductor major*, *semimembranosus*, *semitendinosus*, and *biceps femoris long head* muscles were replaced by fat to a large extent, and the muscles identified with a higher increase in FF were the *biceps femoris short head*, the *vastus intermedius*, and the *vastus lateralis*. The last group (more than 45% baseline mean thigh FF) had 11 individuals, all of them symptomatic. In this group, paraspinal, *adductor major and longus*, *biceps femoris long head*, *semimembranosus*, *semitendinosus*, and *psoas* were replaced by fat and therefore not useful for the follow‐up. In contrast, we identified *vastus lateralis* as the best candidate for the follow‐up. A summary of all these findings is displayed in *Figure* 5.

**Table 9:** Increase in fat fraction depending on visit 0 mean thigh fat fraction

Muscle	Visit 0 FF <15% (*n* = 9)				Visit 0 FF 15‐30% (*n* = 11)				Visit 0 FF 30‐45% (*n* = 6)				Visit 0 FF >45% (*n* = 10)			
	Visit 0	V0‐V3	SRM	MFTR	Visit 0	V0‐V3	SRM	MFTR	Visit 0	V0‐V3	SRM	MFTR	Visit 0	V0‐V3	SRM	MFTR
RF	8.8%	+1.6%	0.87	+1.7%	8.6%	+2.7%	1.56	+2.9%	16.6%	+3.4%	1.44	+3.9%	34.3%	+3.7	0.50	+4.1%
VM	9.8%	+1.2%	0.72	+1.3%	12.9%	+2.8%	0.84	+3.3%	27.9%	+4.1%	1.50	+5.7%	56.8%	+7.3%	0.82	+16.9%
VL	8.1%	+2.5%	1.12	+3.4%	12.5%	+3.9%	1.51	+4.7%	18.4%	+8%	1.28	+9.8%	37.9%	+11.5%	1.52	+20.6%
VI	9.2%	+3.1%	2.32	+3.4%	15.1%	+5.2%	2.51	+6.1%	40.6%	+9%	1.01	+15.2%	66.8%	+5.4%	1.46	+17.1%
BFSH	12.9%	+1.8%	0.55	+2.1%	15.6%	+4.7%	1.73	+5.6%	27%	+9.5%	1.47	+13.1%	44.5%	+6.7%	1.35	+12.8%
BFLH	10.5%	+1.9%	1.01	+2.2%	32.5%	+15.6%	1.51	+23.1%	65.6%	+2.4%	1.23	+7.1%	81.2%	+4.1%	0.90	+24.3%
AM	17.1%	+2%	1.07	+2.5%	75.3%	+3.6%	0.64	+14.6%	84.5%	+3.6%	0.91	+23.1%	91%	+1%	0.76	+8.8%
AL	11%	+2.3%	0.75	+2.5%	30.7%	+5.7%	0.98	+8.3%	42.4%	+2.9%	0.74	+5.1%	75.9%	+4%	0.34	+18.8%
Sa	19.1%	‐0.9%	0.22	‐1.1%	18.6%	+3.3%	0.79	+4.1%	20.7%	+3.8%	1.82	+4.8%	36.5%	+3%	1.28	+5.3%
Gra	15.1%	‐1.4%	0.23	+0.9%	15.9%	+2.6%	1.43	+5.3%	20.1%	+3.3%	1.49	+8.3%	32.1%	+7.7%	0.94	+13.9%
ST	11.5%	+0.7%	0.29	+0.8%	18.3%	+8.8%	0.88	+10.7%	61.6%	+5%	1.29	+13.1%	72.9%	+9.2%	1.03	+36.4%
SM	12.3%	+1.3%	0.31	+1.5%	49%	+8.1%	0.94	+16.1%	80.7%	+1.3%	1.63	+7.1%	86.8%	+2.2%	0.59	+17.1%
Pso	20.7%	+1.4%	0.25	+1.8%	47.6%	+5.9%	0.58	+11.4%	50%	+7.7%	0.8	+15.5%	79.1%	+1.4%	0.46	+6.66%
Ps	38.5%	+5.3%	0.7	+8.7%	78.8%	+3.7%	1.26	+17.7%	85.1%	+0.8%	0.06	+0.5%	87.7%	+0.1%	0.09	+1.81%

Patients were divided in four groups depending on Visit 0 mean thighs fat fraction. Per every muscle, fat fraction at Visit 0 and increase at Visit 3 are shown.

AM, *adductor major*; AL, *adductor longus*; BFSH, *biceps femoris short head*, BFLH, *biceps femoris long head*; FF, fat fraction; Gra, *gracilis*; MFTR, muscle fatty transformation rate; Ps, paraspinal muscle; Pso: *psoas*; RF, *rectus femoris*; VL: *Vastus lateralis*, VM: *Vastus Medialis*, Sa, *sartorius*; SM, *semimembranosus*; SRM, standardized response mean; ST, s*emitendinosus*; VI, *vastus intermedius*; VL, *vastus lateralis*.

## Discussion

In this study, we followed‐up a large cohort of patients with LOPD, both symptomatic‐treated and pre‐symptomatic patients, for a period of 4 years using Dixon quantitative muscle MRI and several clinical assessments including muscle function tests, spirometry, and activities of daily living scales. We detected a significant and continuous increase in muscle fat replacement in symptomatic patients despite being treated with ERT, which correlated to a decrease in muscle strength. In the case of pre‐symptomatic patients, we identified an increase in FF, especially in paraspinal muscles, that was not associated to any change in motor function tests. The study has allowed us to confirm that quantitative muscle MRI is able to identify subtle changes in muscle structure and it can be considered a promising outcome measure for the follow‐up of patients in natural history studies or clinical trials.

Late‐onset Pompe disease is a metabolic disorder characterized by a progressive accumulation of glycogen inside the lysosomes of the tissues. The increasing number of glycogen‐charged lysosomes in the sarcoplasm of the muscle fibres triggers a set of processes that eventually lead to muscle fibre death and replacement by fat and fibrotic tissue.^24^, ^25^ The loss of muscle fibres is related to the muscle weakness of these patients. In recent years, quantitative muscle MRI has been implemented as one of the best candidate biomarkers to follow‐up disease progression in patients with different muscle disorders, such as Duchenne muscle dystrophy, facio‐scapulo‐humeral muscle dystrophy, limb girdle muscle dystrophy 2I, or Pompe disease.^26^, ^27^, ^28^ Most of the previously published studies using qMRI have focused on quantifying the amount of fat and/or water present in the skeletal muscles of the patients. Two‐point or three‐point Dixon, global T2, and ^1^H spectroscopy are the main techniques used for this purpose.^18^ Here, we have used two‐point Dixon, which is a qMRI sequence that can be done in 1.5 Teslas MRIs, available in most of the clinical centres that follow Pompe patients. Our results support the use of Dixon technique as a reliable outcome measure for the follow‐up of patients with LOPD disease. Using this technique, we have shown that skeletal muscle FF increases in symptomatic patients despite ongoing treatment with ERT.

The yearly progression ratio of mean FF, close to 2%, was stable in our cohort. This increase is slightly higher than the one found in previous studies. For example, the group of Professor Carlier performed a retrospective analysis of 14 LOPD patients treated with ERT and found a significant increase of 0.9% in FF of all lower limb muscles during a 1 year period.^19^ However, that study included lower leg muscles, such as *tibialis anterior* or *soleus*, that are commonly less involved in LOPD, probably reducing the final average increase in FF observed. When comparing with other studies, it is important to take into account the acquisition parameters used. For example, in our Dixon sequence, we used a flip angle of 15°that is higher than the one used in previous studies.^19^ A higher T1 weighting, due to the use of a higher flip angle, can lead to an overestimation of the FF, especially for higher FF values.^29^ We have observed considerable variability in the increase of FF from one patient to another, ranging from a 14% increase to less than 1%, suggesting that there are clinical or genetic factors than can influence the progression of the disease. We have observed that the following correlate with a larger increase in FF over a 4 year period: (i) starting ERT at a younger age, suggesting that patients started symptoms earlier; (ii) starting mechanical ventilation early; (iii) worse baseline muscle performance; and (iv) higher baseline fat replacement detected by MRI.

We have also detected significant changes in two function tests: muscle strength and forced vital capacity. These results are in line with other recently published evidence showing decrease of muscle strength in LOPD patients despite ERT treatment.^12^ Moreover, in our study, changes in muscle strength is correlated with increase in FF, which has a clear explanation. Increase in FF comes with a decrease in contractile muscle mass. It is therefore not unusual that those patients in whom FF increased the most had a greater decrease in muscle strength, as has been previously shown in other muscle disorders such as limb‐girdle muscular dystrophy type 2I.^30^ However, decrease in muscle strength did not impact timed test results in our cohort of patients, probably because these tests are not only influenced by muscle strength, but also for other factors such as respiratory involvement, fatigue, muscle pain, or personal motivation.

Quantification of FF using Dixon analysis can be a complicated task that requires a high degree of specialization.^31^ No automatized software is yet available that can distinguish one muscle from the others, and so the analysis has to be performed manually.^32^ In this study, all MRIs were separately analysed by three investigators with considerable experience in this field. We have observed that if anatomical landmarks are well established, the variability in the measurements between investigators is very low. Previous studies have shown that fat replacement is not homogeneous in muscles in patients with muscle diseases, and therefore, analysing one slice only is probably not sufficient to estimate the mean FF of that muscle.^33^ It is therefore necessary to include several slices of every muscle in the analysis, increasing significantly the time needed to complete the study. The identification of single muscles that could act as reporters of the increase in FF is desirable because it could make studies more efficient. Our study allows a description of the typical order in which different muscles are involved, thereby sketching out a hypothetical natural history of fat replacement progression. In our opinion, this is an important step that should be made to select the best muscle to identify changes in FF over a period of time. If a muscle that has already been completely replaced by fat is selected, no changes will be observed as there is no more muscle to lose. On the other hand, if a muscle that is not involved until very late in the progression of the disease is selected, no changes will likely be recorded, even though FF could actually be increasing in other muscles. We have observed that paraspinal muscles are the first muscles involved in LOPD from a radiological point of view, probably leading to the frequently reported symptoms of scoliosis in juvenile patients and lumbar pain or changes in the walking pattern in adults.^34^, ^35^ The *adductor major* muscle is also involved early in the progression of the disease, and in fact, we have observed that these two muscles are almost completely replaced by fat in most of our symptomatic patients.^36^ The muscles of the posterior compartment of the thighs are affected later, and so they can be considered good candidates for monitoring FF progression during intermediate stages of the disease. Finally, *vasti* muscles are involved in more advanced cases. In fact, only two patients in our cohort had clear quadriceps weakness leading to difficulties walking downstairs or standing. *Vastus lateralis* is, in the light of our results, a good candidate for monitoring FF progression in patients that are at an advanced clinical stage.

We had the opportunity to study four patients before and after starting ERT. Interestingly, we observed that ERT decreased the rate of increase of FF from 3% to 1.7%, which is a value close to the mean observed in the whole cohort, suggesting that ERT with Myozyme® is effective in slowing down the progression of muscle degeneration in Pompe disease. These results are in accordance with those previously reported by the group of Professor Carlier. This group reported that increase in FF was lower in patients treated with ERT than in those not treated after 1 year of follow‐up (19).

Through our study, we realized that Dixon has some clear limitations in the study of pre‐symptomatic Pompe patients. Increase in mean thigh FF was only significant in those pre‐symptomatic patients older than 25 years old but not in the younger patients whose age ranged from 8 to 21 years old. This can be related to the fact that the process of muscle degeneration and fatty replacement was not active in younger patients. However, we identified two different situations that should be discussed and analysed in further studies. We already identified fat replacement in paraspinal and *adductor major* muscles of some pre‐symptomatic patients at baseline visit, suggesting that the process of muscle degeneration progresses very slowly in Pompe, and is subclinical in some patients, until a moment in which patients experience muscle weakness. However, we identified two young patients that developed muscle weakness, one during the study, and the other just at the end, in which increase in FF was not detected despite those patients complaining of worsening muscle weakness. In the first case, we observed mild psoas and glutei weakness in a 19‐year‐old girl. At that moment, her MRI did not show muscle fat replacement. The second case was a 12‐year‐old boy who developed scoliosis and glutei weakness 6 months after Visit 3 (end of the study). We performed a new MRI that did not show muscle fat replacement. It is therefore probable that in some cases, the accumulation of glycogen‐charged lysosomes, or the presence of free glycogen in the sarcoplasm, disrupts muscle fibre contraction, leading to muscle weakness before inducing necrosis of the fibre and fatty substitution.^37^ In these cases, Dixon may not be useful, because there are no changes in fat tissue content. Therefore, a combination of Dixon with a muscle MRI sequence able to identify glycogen in the muscle fibres of weak muscles may more informative in this subset of patients. Muscle glycogen can be identified and quantified using some specific muscle MRI sequences, such as GlycoCEST or (13)C‐MR spectroscopy, but this technology is not routinely available on standard clinical scanners.^38^, ^39^ An increased signal intensity in Short Tau Inversion Recovery (STIR) sequence in muscles from LOPD patients has been described, suggesting that this is related to the presence of water molecules retained by glycogen.^40^ In a similar way, increase in the water T2 signal in some muscles was identified in LOPD patients, which correlated with a higher increase in FF after 1 year follow‐up, suggesting that water T2 could be a potential marker of disease activity.^19^ However, increased signal intensity in STIR or changes in water T2 is not specific and can be observed in muscle fibre necrosis, inflammation, or denervation.^41^ In our opinion, for Pompe patients, studying MRI sequences that are able to identify changes related to the accumulation of glycogen is likely to be especially useful for the study of juvenile patients who are experiencing muscle symptoms.

Our results support the use of muscle MRI in the decision‐making process in patients with Pompe disease. We have shown that a high value in FF in skeletal muscles at baseline is associated with a quicker progression of the disease. Moreover, there is a correlation between increase in FF and changes in muscle function tests, which translates the clinical importance of the radiological findings. As fat replacement is an irreversible process, we suggest that muscle MRI should be part of the initial assessment of patients with LOPD and taken into account in deciding when to start treatment in pauci‐symptomatic patients.

To conclude, the results of our longitudinal study (i) establish the rate of annual progression of fat replacement in LOPD patients treated with ERT with Myozyme; (ii) pinpoint clinical factors influencing the increase in muscle FF; and (iii) show the clinical relevance of the changes observed in muscle MRI.

## Contributions of authors

CNP, JL, SS, PM, IB, IP, EM, JSG, AMN, II, and JDM carried out the concept and design of the study. CNP, JAP, JL, IB, IP, EM, and AAJ performed the acquisition and analysis of data. CNP, JAP, JL, SS, PM, AAJ, JSG, AMN, II, and JDM drafted of the manuscript. SS performed the acquisition of data. II and JPM obtained funding for the study. Spanish Pompe Study Group: see supplemental Table S1.

## Conflicts of interest

The company has neither reviewed the data nor suggested changes in any of the conclusions of the paper. Claudia Nuñez‐Peralta, Jorge Alonso‐Pérez, Jaume Llauger, Sonia Segovia, Paula Montesinos, Izaskun Belmonte, Irene Pedrosa, Elena Montiel, Alicia Alonso‐Jiménez, Javier Sánchez‐González, and Antonio Martínez‐Noguera have no conflict of interest.

## Supporting information


**Table S1** Members of the Spanish Pompe Study Group Placement
