# Supplementary material for: Follow‐up of late‐onset Pompe disease patients with muscle magnetic resonance imaging reveals increase in fat replacement in skeletal muscles
Source: J Cachexia Sarcopenia Muscle. 2020 Mar 4;11(4):1032–46. doi: 10.1002/jcsm.12555 (PMC7432562; doi:10.1002/jcsm.12555)
Supplement: Supplementary file 1 — Table S1 Members of the Spanish Pompe Study Group Placement [file JCSM-11-1032-s001.docx]

| **Name** | **Institution** |
| --- | --- |
| Barba-Romero, M. A. | Hospital General de Albacete |
| Barcena, J. | Hospital Universitario Cruces, Baracaldo |
| Carrasco-Rozas, A. | Hospital de la Santa Creu i Sant Pau, Barcelona |
| Cazorla, M. R. | Hospital Puerta de Hierro, Majadahonda |
| Creus, C. | Hospital Virgen de las Nieves, Granada |
| Coll-Cantí, J. | Hospital Germans Tries i Pujol, Badalona |
| De Luna, N. | Hospital Santa Creu I Sant Pau, Barcelona |
| Díaz, M. | Hospital de Cabueñes, Gijón |
| Domínguez-González, C. | Hospital 12 de Octubre, Madrid |
| Fernández-Simón, E. | Hospital de la Santa Creu i Sant Pau, Barcelona |
| Figueroa-Bonaparte, S. | Hospital |
| Gallardo, E. | Hospital de la Santa Creu i Sant Pau, Barcelona |
| Grau, J.M. | Hospital Clinic, Barcelona |
| López de Munáin, A. | Hospital Universitario Donostia, San Sebastián |
| Martínez-García, F.A. | Hospital Clínico Universitario Virgen de la Arrixaca, Murcia |
| Morgado, Y. | Hospital Universitario Virgen de Valme, Sevilla |
| Morís, G. | Hospital Universitario Central de Aturias, Oviedo. |
| Muñoz-Blanco, M.A. | Hospital Gregorio Marañón, Madrid |
| Nascimento, A. | Hospital Sant Joan de Deu, Barcelona |
| Olivé, M. | Hospital de Bellvitge, Barcelona |
| Paradas, C. | Hospital Virgen del Rocío, Sevilla |
| Querol, L. | Hospital de la Santa Creu i Sant Pau, Barcelona |
| Robledo-Strauss, A. | Hospital Juan Ramón Jiménez, Huelva |
| Rojas-García. R. | Hospital de la Santa Creu i Sant Pau, Barcelona |
| Rojas-Marcos, I. | Hospital Virgen del Rocío, Sevilla |
| Turon, J | Hospital de la Santa Creu i Sant Pau, Barcelona |
| Salazar, J.A. | Hospital Regional Universitario, Málaga |
| Suárez-Calvet, X. | Hospital de la Santa Creu i Sant Pau, Barcelona |
| Usón, M. | Hospital de Son Llátzer, Palma de Mallorca |
